# Evaluation of the Performance of Dual Polyelectrolyte Systems on the Re-Flocculation Ability of Calcium Carbonate Aggregates in Turbulent Environment

**DOI:** 10.3390/polym8050174

**Published:** 2016-04-29

**Authors:** Maria G. Rasteiro, Fernando A. Garcia, David Hunkeler, Ineide Pinheiro

**Affiliations:** 1 Research Centre on Chemical Processes and Forest Ptoducts—CIEPQPF, Chemical Engineering Department, Coimbra University, 3030-790 Coimbra, Portugal; fgarcia@eq.uc.pt (F.A.G.); pinheiro_468@hotmail.com (I.P.); 2 AquaTECH Specialities, CH-1237 Avully, Switzerland; david.hunkeler@aquatech-water.com

**Keywords:** flocculation, re-flocculation, dual polyelectrolytes, laser diffraction spectroscopy, papermaking

## Abstract

Flocculation can be used in turbulent environments resulting in floc breakage due to shearing. The degree of re-flocculation relates directly to product quality and process efficiency. This study aimed at looking for alternatives to improve the re-flocculation ability of aggregates when polyelectrolytes (PEL) are used as flocculation agents. Moreover, because branched PEL have proved previously to lead to high flocculation efficiencies, the work presented focus on the improvement of the re-flocculation ability of branched PEL. Thus, a selection of branched polymers were used primarily as flocculation aid and after flocs break up a linear polymer was added to the system in order to improve re-flocculation. Different mixtures were tested with the objective to try to induce, during re-flocculation, complementary flocculation mechanisms, favoring the patching mechanism. Re-flocculation improved significantly with this strategy. Laser Diffraction Spectroscopy was used to monitor the flocculation and re-flocculation processes supplying information about the floc size and structure. Since inorganic materials, namely bentonite, have been widely used to improve the re-flocculation capacity of polyelectrolytes, the results of using dual polyelectrolyte systems were compared with the effect of adding bentonite to the system.

## 1. Introduction

Polyelectrolytes (PEL) are widely used to achieve efficient solid-liquid separation in many industries. In the case of papermaking, flocculation is the most important phenomena of the wet-end stage since it affects process efficiency and the quality of the final product [1,2,3,4,5,6]. Studies on the wet-end chemistry have established that depending on the retention systems used, aggregation of the particles can occur by charge neutralization, patching, bridging or complex flocculation mechanism [1,2]. However, to control the flocculation process it is necessary to know and understand how chemical additives act during the whole process. Moreover, in many industrial processes, as for instance in papermaking and food industries, flocculated suspensions are subjected to high shear forces. In the case of the wet end of a paper machine where turbulence is high to favor the uniform formation of the paper sheet [7], floc break up due to shearing and partially re-flocculate when the shear forces decrease [7]. Therefore, the degree of re-flocculation does have a strong influence on both product quality and process efficiency. When polyelectrolytes are used to induce aggregation of a suspension, the floc strength and the re-flocculation capacity depend on the predominant flocculation process [3,8,9,10,11,12]. For example, polymer detachment from particles surface can result also in chain degradation and original bonds become difficult to reform reducing the efficiency of aggregation between floc fragments. If re-flocculation is difficult, additives adjoined to the system may be lost during the process, which is, of course, undesirable.

Various processes occur simultaneously during flocculation: adsorption of polymer molecules at the particles surface; re-arrangement (or re-conformation) of adsorbed polymeric chains; collisions between destabilized particles to form aggregates (floc); and break-up of floc [10,13,14]. The importance of each process depends on the flocculant characteristics, such as chain architecture, molar mass, charge density; on the characteristics of the suspended particles; on the characteristics of the suspending medium; and, finally, on the PEL concentration, contact time and turbulence intensity, among others [14,15]. The macromolecular characteristics of the polyelectrolyte determine its conformation when adsorbed on the particle surface and, therefore, the predominant flocculation mechanism [15]. In general, if molar mass is high and the charge density is low the flocculation process is dominated by bridging bonds [10]. When the charge density is high, the bridging capability is reduced because there is a tendency for the polymer chains to adopt a flatter conformation on the particle surface, which results in the formation of cationic patches that attract the polymer free, oppositely charged surfaces of other particles [15]. On the other hand, the introduction of branches in the polymer chain can alter the PEL conformation on the particle surface and, again, influence the aggregation mechanism [16]. Branched polymers acquire a more coiled conformation in solution, requiring a lower amount of polymer for surface coverage, still, this can render re-flocculation more difficult since less active polymer will be available after breakage [17].

As mentioned above, another important feature is the floc resistance. The stability of the floc depends on the nature of the interaction between particles and on the floc density [7] and re-flocculation depends on the predominant flocculation mechanism [7]. When bridging is the main flocculation mechanism floc are stronger. However, the more resistant are the floc the more difficult is re-flocculation when they break [18]. Of course higher shear rates are needed when dealing with more resistant flocs and, when the shear force increases, the tails and loops of high molecular weight polymers are broken. Therefore, when the shear force decreases thereafter, the possibility of re-flocculation by bridging decreases and re-flocculation takes place rather through the patch mechanism [3,18,19]. In the case of flocculation induced by patching, the effect of shear forces on the polymer is lower but, if the polymer is re-conformed within the diffuse layer, the interactions with other particles decrease. Hence, re-flocculation, though easier, may also happen to a lower extent than the original flocculation degree [19].

This study aimed at looking at alternatives to improve the re-flocculation capacity of aggregates when PEL are used as flocculation agents. Moreover, because branched PEL have proved previously to lead to high flocculation efficiencies [11,16], the work presented focus on the improvement of the re-flocculation ability of branched PEL through addition of a secondary additive. In fact, when using the branched polymers, after a shearing stage the re-flocculation capability of the broken floc is very small because aggregation takes place mainly by the bridging mechanism. Different mixtures were tested: branched PEL mixed either with a linear polymer of similar molar mass (MW) or with a linear polymer with the same charge density and composition but with lower MW; alternatively, the branched PEL was mixed with a linear PEL of similar MW but higher charge density. The objective was to try to induce, during re-flocculation, complementary flocculation mechanisms, favoring patching. It has been proved that re-flocculation is improved when the patching mechanism is favored [20]. Dual polyelectrolyte systems have been tested previously, with success, in papermaking applications [21,22], even if re-flocculation was not evaluated in those studies.

Since inorganic materials have been widely used to improve the re-flocculation ability of polyelectrolytes, namely bentonite [23], the results of using dual polyelectrolyte systems will be compared with the effect of adding bentonite to a branched PEL, after floc breakage.

In this study, the flocculation and re-flocculation performance was investigated using laser diffraction spectroscopy (LDS), following previous studies from the authors [19,23].

## 2. Materials and Methods

### 2.1. Materials

The flocculation tests were carried out using a commercial scalenohedral precipitated calcium carbonate (PCC) suspension supplied by OMYA, Oftringen, Switzerland. The PCC suspension (median size 2.05 μm, suspension pH 8.5, zeta potential −32 mV) was prepared at 1% (*w*/*w*) in distilled water. In order to obtain a good dispersion of the particles, the suspensions were first magnetically stirred for 20 min and then submitted to sonication at 50 kHz during 15 min. In this study, only branched cationic polyacrylamides (C-PAM), with 4 branches per chain, all copolymers of acrylamide (AM) and acryloyloxyethyltrimethyl ammonium chloride (Q9), developed and supplied by AquaTECH Specialities, Avully, Switzerland [24,25,26], with a cationic monomer content between 45% and 34% *w*/*w*, as determined by argentometric titration (Metrohm, Herisau, Switzerland), were used as initial flocculation aids (Table 1), since the objective was to look at ways to improve the re-flocculation ability of branched PEL. In this table the “+” in the polymer reference indicates the number of branches in the polymer chain, which correspond to the equivalent of crosslinker added during the synthesis. Different linear C-PAMs, also varying in molar mass and charge density, as summarized in Table 1, were tested as re-flocculation aids, as an alternative to the use of inorganic microsized particles such as bentonite. The linear PEL had either the same charge density as the equivalent branched ones or a higher charge (BHMW). In Table 1 the following nomenclature was adopted when naming the polymers: B refers to charge density ≈ 80%, E ≈ 40% and F≈ 35%; as for the molecular weight, HMW refers to very high molecular weight, 1 to high molecular weight and 2 to medium molecular weight, The intrinsic viscosity of the polymers was determined in 0.05 M NaCl solution by dilution viscometry [27], using an automatic capillary viscometer Viscologic TI1 (Sematech, Nice, France). Molecular weight of the linear polymers was measured also with polymer solutions prepared in 0.05 M NaCl solution, by analytical ultra-centrifugation, using an OPTIMA XL-I (Beckman Coulter, Brea, CA, USA) [28]. The branched polymers have got, according to the supplier, molecular weight identical to the equivalent linear ones.

Flocculant solutions were prepared everyday with distilled water at 0.1% (*w*/*w*). Bentonite (particle size 5.7 μm) was also used as re-flocculation aid, to enable comparison with the dual polymers systems.

### 2.2. Methodology

PCC flocculation was monitored by measuring the aggregates size using laser diffraction spectroscopy (LDS) in a Malvern Masterziser 2000 (Malvern Instruments, Malvern, UK), according to the procedure developed previously [17,19]. All tests were repeated at least three times and the maxium deviation in the median of the size distribution, for each time instant, was always lower than 3%. The PCC suspension was added to 700 mL of distilled water in the equipment dispersion unit until a certain, fixed level of obscuration was obtained, corresponding to an average PCC concentration of 0.1% (*w*/*w*) and the tests were carried out setting the pump speed to 1400 rpm (312 s*^−^*^1^). During the whole process, the flocculation vessel was stirred mechanically using the sample unit facilities of the Malvern Mastersizer 2000 equipment.

As obscuration decreases pronouncedly during the flocculation test, due to floc growth, the tests have to be initiated with a higher obscuration than the usual procedure, without compromising signal quality. This value was previously optimized [19]. Moreover, during the entire flocculation process, obscuration was always kept above 5% to assure a good signal quality [19]. The concentration of the branched polymer added initially was optimized previously [17]. The criteria was to consider optimal the concentration which lead to larger flocs and fastest flocculation kinetics. Regarding the bentonite particles they had a median size of 5.7 μm (measure by LDS) and the bentonite suspension was prepared at 2% (*w*/*w*) in distilled water. The concentration of bentonite in the re-flocculation tests was kept at 2.5 mg/g PCC. This value was optimized in a previous study [23]. Excess bentonite is detrimental for the flocculation process. To obtain the flocculation kinetics curve, the predetermined amount of flocculant was added at once to the suspension and the floc size distribution was measured every minute during 14 min. The particle size distribution of the PCC was measured before adding the flocculant to the suspension.

The flocs resistance was evaluated by submitting the flocs to sonication at 20 kHz during 30 s. Intensity and sonication time were tuned previously [20] to better reproduce shearing in industrial processes. After the shearing test the shear force was restored to the initial value to allow for the re-flocculation process to take place, which was then monitored for 6 min. Stirring in the vessel, during the re-flocculation stage, was the same as during the initial flocculation step. As referred, branched polymers were used primarily as flocculation aid and two strategies were followed for addition of the second PEL: firstly the two polymers were added at the start of the flocculation process, and then, after stabilization, flocs were forced to break and allowed to re-flocculate; secondly, flocculation started using only the branched PEL and only after flocs break up the linear polymer was added. When using as re-flocculation additive the bentonite suspension, the inorganic particles were also only added after flocs break-up. The reported values of the median particle size (dp50) represent an average of at least four replications.

**Figure 1:**
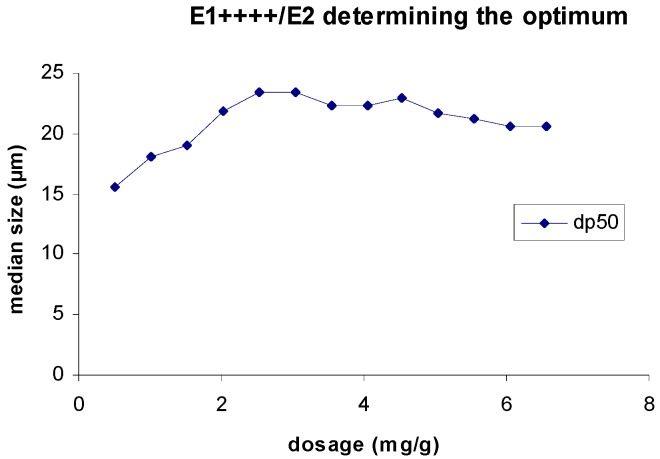
Determination of the optimum dosage of E2 for re-flocculation after floc break-up when E1++++ (8 mg/g PCC) is used as initial flocculant.

When adding the linear polymer after flocs break-up, and in order to evaluate the most suitable range of concentration for detailed testing, a preliminary test was conducted. In this test, the linear polymer was continuously added, after flocs breakage, and the value of the median flocs size, thirty seconds after polymer addition, was registered. This procedure went on till a maximum on the curve of dp50 *versus* the linear polymer concentration was reached. Figure 1 presents one of these curves, for the re-flocculation of the branched E1++++ with the linear polymer E2. In fact, when the linear polymer is in excess the re-flocculated flocs size stops increasing. From this curve we have identified the optimal range of E2 concentration (which maximizes re-flocculation) to lie between 2 and 4 mg polymer/g PCC. This procedure was adopted for all the polymer mixtures tested and Table 2 summarizes the range of concentrations tested for each pair branched/linear polymer. In the following tests the linear PEL was added at once, after flocs break-up, for the previously identified optimal range of concentrations.

LDS does also allow us to calculate the mass fractal dimension of the floc (*d*_F_) as a function of time [10,17]. Mass fractal dimension provides a mean of expressing the degree to which primary particles fill the space within the nominal volume occupied by an aggregate, according to Equation (1) (for solid non-porous particles *d*_F_ = 3 and for porous particles 1 < *d*_F_ < 3) [29].

(1) $$m(R)\propto \;R^{d_{F}}$$

where *m* is the mass of the aggregate of radius *R*.

For systems where flocculation is very pronounced the individual particles can be considered to follow the Rayleigh-Gans-Debye approximation [29] but, for the secondary aggregates, resulting from the aggregation of primary ones, the so called scattering exponent, *S*_E_, corresponding to scattering at larger length scales [17,29], should be computed from the scattering pattern. The kinetics of the floc structure evolution was computed off-line, through the mathematical processing of the scattering matrix.

## 3. Results and Discussion

### 3.1. Re-Flocculation with Dual Polyelectrolyte Systems

The re-flocculation ability of the flocs produced only with the branched polymers is very small or practically non-existent (Table 3). If flocs formed by bridging mechanism break up, the polymer can also recomform and eventually degrade, specially if attached to the particles surface at several points, and the re-flocculation process becomes more difficult [3,30].

Re-flocculation tests using dual polymer systems were conducted in two different ways: firstly the two polymers (branched and linear) were added together, in different relative proportions, to the PCC suspension, at the beginning of the flocculation process; secondly, the linear polymer was only added after flocs break up, different concentrations, in the range previously optimized, having been tested.

**Figure 2:**
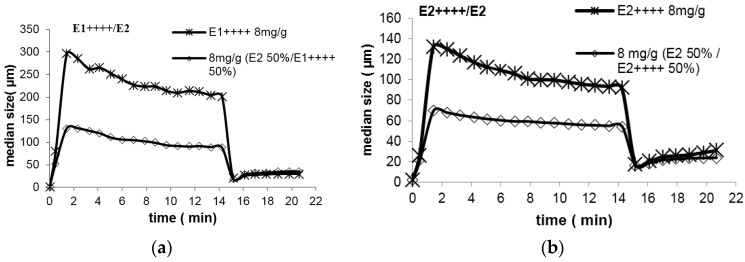
Re-flocculation with mixture (8 mg/g PCC) of branched and linear polymer (50/50% weight) added simultaneously at the beginning of flocculation: (**a**) E1++++/E2 and (**b**) E2++++/E2.

Figure 2 shows two examples of results achieved with the first strategy (simultaneous addition of the branched and the linear polymers), for a relative ratio of 50% (weight) of each polymer, for the pairs E1++++/E2 and E2++++/E2. As can be observed from this figure, re-flocculation did not improve when the dual system is added at once at the beginning of the flocculation process, if compared with the situation where only the branched polymer is used. This conclusion is common to all the pairs of branched/linear polymers tested in this way, and for all the proportions considered (75/25, 50/50 and 25/75 ratios of branched to linear polymer), independently of the type of the linear polymer added. It is also apparent from Figure 2 that, if the molecular weight of the branched PEL is higher (Figure 2a), larger flocs are obtained, as expected.

**Figure 3:**
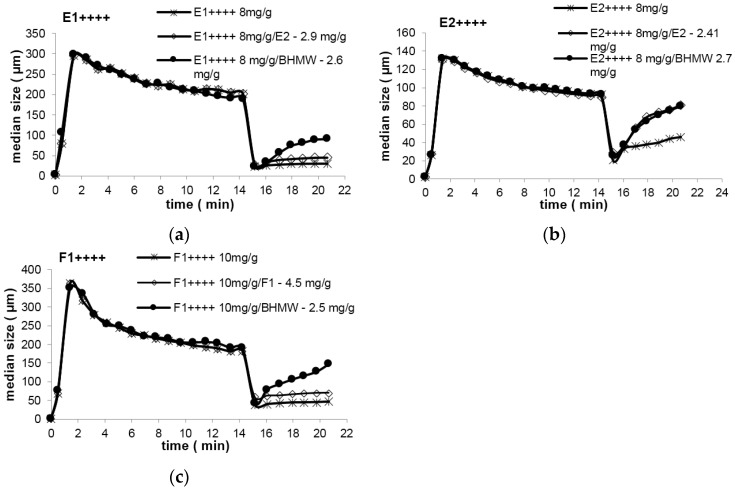
Re-flocculation with addition of linear polymer after flocs break-up: (**a**) E1++++/E2 and E1++++/BHMW; (**b**) E2++++/E2 and E2++++/BHMW; (**c**) F1++++/F1and F1++++/BHMW.

Figure 3 presents the results obtained (flocculation and re-flocculation kinetic curves) when the linear polymer is added after flocs break-up. Table 3 summarizes also those results, presenting the values of the re-flocculation percentage for all the situations tested, which is calculated as the ratio between the difference of the flocs size after re-flocculation and after breakage and flocs size before breakage. In general, it was possible to find a concentration of the linear polymer for which re-flocculation increased when compared with the situation where only the branched polymer was used.

Additionally, it is possible to conclude that the addition of a linear polymer with a high charge density favors re-flocculation. This is more notorious when branched polymers of higher molecular weight are used as initial flocculant (E1++++ and F1++++). When E2++++ (lower molecular weight) is used as initial flocculant, similar degrees of re-flocculation were achieved using either the linear E2 (charge density similar to the one of the branched polymer) or the linear BHMW (higher charge density) as re-flocculation additives. These results suggest that re-flocculation is enhanced if bridging and patching mechanisms coexist in the process. When flocculation starts based on the bridging mechanism, as happens when high molecular weight polymers are used, re-flocculation can only occur if the patching mechanism is induced later, during the re-flocculation process which takes place after breakage. As higher charge density of the polymer favors the patching mechanism, re-flocculation was better when BHMW was used in combination with either E1++++ or F1++++ which, considering their molecular weight, work based on the bridging mechanism. Even if the addition of E2 (lower molecular weight), acting also based on the patching mechanism, did also improve re-flocculation, the ability of this polymer to flocculate the broken aggregates is not as good as when a polymer of higher charge density is used, since it must produce smaller patches. In the case of E2++++, since the branched polymer has a lower molecular weight, which must lead initially, in the first stage of flocculation, to a combination of bridging and patching mechanisms, the influence of the linear polymer charge density on re-flocculation is not so noteworthy, and both E2 and BHMW lead to similar re-flocculation percentages.

The hypothesis of re-flocculation being enhanced when the patching mechanism is favored is confirmed if we analyze further the results obtained when F1++++ is used as initial flocculant. F1++++ has a lower charge density and a very high molecular weight (in the same range of E1++++) thus, this polymer will act based only on the bridging mechanism. F1++++ is, in fact, the polymer with the lowest re-flocculation ability when used alone, as is typical of flocs produced through the bridging mechanism. Therefore, when a linear polymer with equal charge density and similar molecular weight is used as re-flocculation aid (F1), re-flocculation is not improved since the conditions to induce patching are not created. However, if a linear polymer with a considerable higher charge density is used (BHMW), re-flocculation is improved dramatically. The best re-flocculation results, when we consider all the tests conducted, are indeed obtained for the pair F1++++/BHMW, since patching will be most favored in this case, during the re-flocculation stage.

In summary, adding a linear PEL of lower molecular weight or higher charge density to the aggregates produced with the branched PEL after they are forced to break, improves re-flocculation. Moreover, there is an optimum value for the concentration of the linear PEL, which maximizes re-flocculation.

**Figure 4:**
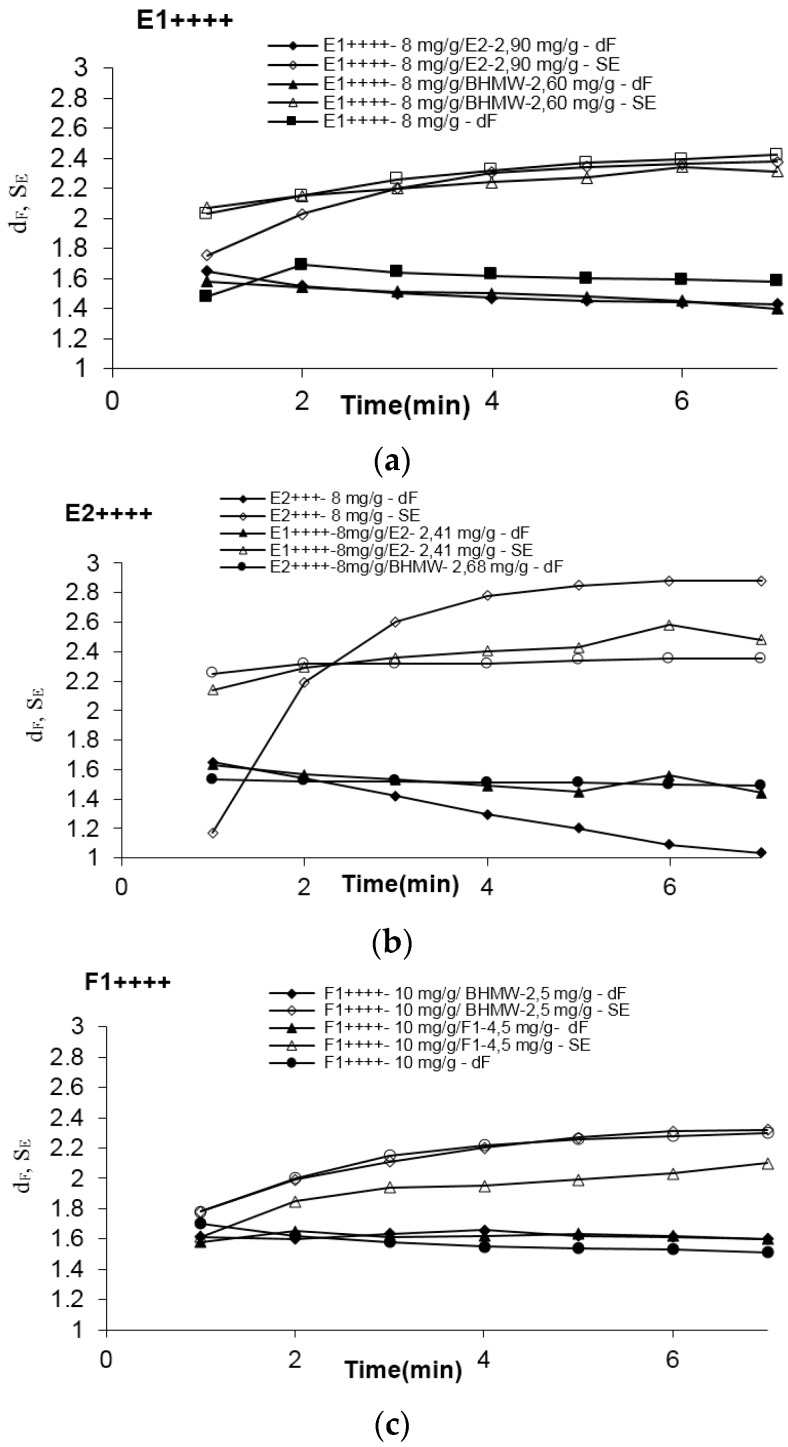
Evolution with time of mass fractal dimension and scattering exponent of the re-flocculated flocs with addition of linear polymer after floc break-up: (**a**) E1++++; (**b**) E2++++; (**c**) F1++++.

Figure 4 presents the evolution with time after breakage, of both the scattering exponent, referring to the structure of the secondary aggregates, and the fractal dimension of the flocs. Considering the size of the flocs and what has been discussed previously [10,17], the important parameter which should be considered to describe the flocs structure is the scattering exponent, which refers to scattering at large length scales, that is to scattering from secondary aggregates.

Analyzing the graphs in Figure 4 it is obvious that during the re-flocculation process flocs become more compact (*S*_E_ increases). Table 3 includes also the values of *S*_E_ at the end of the re-flocculation process, for the different tests, as well as the values of *S*_E_ at the end of the first stage of flocculation, when only the branched polymer is present in the system. For all the situations tested flocs become more compact after break up and re-flocculation, independently of the re-flocculation aid used, than at the end of the primary flocculation stage. This is in-line with previous studies [23].

The main conclusion which can be extracted from these results is that, except for the case where a low charge density polymer was used as initial flocculant (F1++++), the flocs at the end of the re-flocculation step present all a similar structure, that is a similar degree of compaction (similar *S*_E_ values), independently of the linear polymer which is added to enhance re-flocculation. Therefore, the addition of the linear PEL is not detrimental for flocs structure despite improving re-flocculation. On the other hand, the flocs obtained with F1++++ (lower charge density) are initially, at the end of the flocculation stage, less compact, which is compatible with the use of a lower charge density polymer. When a linear low charge density polymer with similar molecular weight (F1) is used as re-flocculation aid, the flocs become even more open than when only F1++++ was used during the whole process, since bridging is the mechanism favored also during re-flocculation. On the contrary, when a high charge density polymer (BHMW) is used as re-flocculation additive the re-flocculated flocs become much more compact, since the patching mechanism is now favored during re-flocculation. Overall, when using the pair F1++++/BHMW it is possible to improve re-flocculation substantially (see Table 3) and to obtain, simultaneously, structured flocs with a high compactness.

Looking now at the *S*_E_ values obtained for the mixtures where E2++++ is present, it is also apparent that these floc are always more compact, which agrees with the use of a lower molecular weight polymer as initial flocculant.

In summary, it has been demonstrated that when the patching mechanism is favored during re-flocculation more compact flocs are obtained. Additionally, the results show that by favoring this mechanism during this step re-flocculation ability is enhanced. The analysis of the floc structure after re-flocculation (Table 3) supports that for re-flocculation to be significant it is necessary to favor the patching mechanism during this stage.

### 3.2. Re-Flocculation Induced by Bentonite

Table 3 presents also the re-flocculation percentage for the tests where bentonite was used as re-flocculation aid. Previous studies from the authors of [23] proved that bentonite is a very effective re-flocculation additive for calcium carbonate particles. In that study it has also been proved that excess bentonite is detrimental for re-flocculation. Thus, the bentonite concentration optimized in the previous study (2.5 mg bentonite/g PCC) was used in the tests presented here.

Looking at Table 3 it can be concluded that bentonite was always a more effective re-flocculation aid than the dual polymers systems. Bentonite forms complexes with the polymer enhancing the bridging capacity of the polymer, which is altogether more important when this capacity is reduced due to breakage of the polymer chains as a consequence of floc shearing, and subsequent polymer degradation. However, in both the cases where E2++++ or F1++++ were used as initial flocculants the differences in the re-flocculation efficiency obtained with the two strategies aforementioned (addition of inorganic particles or of a linear high charged polymer) are not very substantial. Again, when E2++++ was used as primary flocculant a lower re-flocculation efficiency was achieved, which can be attributed to the flocculation mechanism associated to this polymer in the first stage of flocculation, which combines bridging and patching due to its lower molecular weight.

Floc structure is always more compact at the end of re-flocculation when bentonite is used (see Table 3). When E1++++ was used as initial flocculant, bentonite was much more efficient than the dual polymers system. In this case a significantly higher re-flocculation percentage was achieved with bentonite than when either E2++++ or F1++++ were used as initial flocculants. This fact agrees with a previous study [23] where it was shown that bentonite is less effective when lower charge density polymers are used as initial flocculants. This must be related to the lower polymer degradation which must occur during floc break-up in the case of E1++++ when compared with F1++++ (polymers with similar molecular weight but E1++++ possessing a higher charge density), which may favor the interaction of the polymer with bentonite after floc and polymer chains are broken during the breakage stage. Looking at the structure of the secondary floc produced with bentonite (*S*_E_ values in Table 3), they are, in general, more compact than either the re-flocculated aggregates produced only with the branched polymers or with dual polymers systems. The charge density of the initial polymer did not show any significant influence on the structure of the final floc produced with bentonite.

In summary, it can be stated that it is possible to improve the re-flocculation ability of branched polyelectrolytes, even if they have on their own a poor re-flocculation ability, which is an important objective considering that the structure of the floc produced with these polymers is the most adequate for certain industrial processes [31]. To overcome their low re-flocculation capacity it is possible to add a second PEL of similar chemistry but with a linear chain structure and, preferably, with a higher charge density. Alternatively, inorganic particles, as for instance bentonite, can be added to the system, which usually lead to an increased performance regarding re-flocculation. However, in some processes it is not convenient, considering the targeted final product characteristics, to add a second type of inorganic particles to the system. Thus, dual polymeric systems tuned to have the capability to induce aggregates re-flocculation can be a good solution. Additionally, bentonite can easily induce over re-flocculation, leading to too large and open aggregates, which can be a problem for some processes and products [23].

## 4. Conclusions

Re-flocculation of calcium carbonate flocs produced with branched polymers of different charge density was evaluated. The study focused on primary flocculation induced by branched polymers, due to their good performance demonstrated in previous flocculation studies [31], regarding both flocculation kinetics and floc structure. However, even if these polymers exhibit a good flocculation performance, they have a very low re-flocculation ability under turbulent environment, which is detrimental for their application in certain processes or products. To improve re-flocculation of these polymers, dual systems, either adding a linear polyelectrolyte of similar chemistry to the branched one, or mixing the branched polylectrolyte with inorganic microparticles (bentonite), were tested. The main conclusions from this study are summarized next.

1. Re-flocculation of floc formed with branched PEL can be significantly improved by using dual systems;
2. Re-flocculation is only improved if the re-flocculation additive is added after floc breakage;
3. When the flocs are initially formed based on the bridging mechanism, re-flocculation is most improved if the patching mechanism is induced during the re-flocculation process, that is, if both mechanisms coexist during that stage. Thus, if a linear, high charge density polymer is used as re-flocculation additive, after floc break-up, the re-flocculation process is more effective;
4. Floc become more compact after re-flocculation with the dual polymers system, confirming that the patching mechanism was induced during this stage;
5. The inorganic particles lead, in general, to higher re-flocculation efficiencies and more compact floc. However, we must be aware that for certain processes/products it is not convenient the addition of a second type of inorganic material, considering the targeted final product characteristics.

Thus, to conclude, tuning dual polymeric systems to have the capability to induce re-flocculation of aggregates in turbulent environment, can be an interesting solution for certain processes. Finally, the LDS technique could be used with success to evaluate the influence on re-flocculation of aggregates, in turbulent environment, of different re-flocculation additives.

## Figures and Tables

**Table 1:** Flocculants characteristics.

Initial branched polymer alpine floc^™^	Intrinsic viscosity—IV ^a^ (mL/g)	Cationic content (*w*/*w* %)	Optimum dosage of branched PEL (mg PEL/g PCC)	Linear re-flocculation polymer alpine floc^™^	Intrinsic viscosity—IV ^a^ (mL/g)	Molecular weight (g/mol) × 10^−6^	Cationic content (*w*/*w* %)
E1++++	1,772	42.8	8	E2	1,550	0.13	47.3
E2++++	977	43.2	8	F1	1,399	4.2	35.2
F1++++	914	34.4	10	BHMW	3,050	7.2	80.0

^a^ Schulz-Blaschke in 0.05 M NaCl [27].

**Table 2:** Range of concentrations of linear polyelectrolytes tested in re-flocculation when the linear PEL is added after floc break-up.

Pair of branched/linear PEL	E1++++/E2	E1++++/BHMW	E2++++/E2	E2++++/BHMW	F1++++/F1	F1++++/BHMW
Range of concentration of linear PEL (mg PEL/g PCC)	1–4	2.5–5	0.95–4	1–4.5	2.5–6.5	2.5–4.5

**Table 3:** Summary of flocculation and re-flocculation indicators.

Primary flocculant concentration (mg/g PCC)	Re-flocculation aid (mg/g PCC)	*d*_50_ after 1st flocculation stage (µm)	*d*_50_ after re-flocculation (µm)	*S*_E_ after 1st flocculation stage	*S*_E_ after re-flocculation	Re-flocculation %
E1++++ (8 mg/g)	–	198.7	40.2	2.27	2.42	8
E1++++ (8 mg/g)	E2 (2.9 mg/g)	198.1	41.2	2.28	2.38	14
E1++++/E2 (50/50) 8 mg/g	–	54.3	24	–	2.37	14
E1++++ (8 mg/g)	BHMW (2.6 mg/g)	191.2	95.5	2.27	2.33	40
E1++++ (8 mg/g)	Bentonite (2.5 mg/g)	191.8	159.1	2.26	2.59	70
E1++++ (8 mg/g)	Bentonite (25 mg/g)	191.8	80.5	2.26	2.0	29
E2++++ (8 mg/g)	–	123.2	60.1	2.38	2.53	26
E2++++ (8 mg/g)	E2 (2.41 mg/g)	120.5	96.1	2.39	2.48	48
E2++++/E2 (50/50) 8 mg/g	–	88.0	34.9	–	–	18
E2++++ (8 mg/g)	BHMW (2.7 mg/g)	120.2	94.0	2.34	2.37	46
E2++++ (8 mg/g)	Bentonite (2.5 mg/g)	128.3	120.1	2.38	2.78	62
E2++++ (8 mg/g)	Bentonite (25 mg/g)	128.3	67.1	2.38	2.1	21
F1++++ (10 mg/g)	–	199.3	45.9	2.19	2.30	6
F1++++ (10 mg/g)	F1 (4.5 mg/g)	197.6	69.9	2.17	1.96	7
F1++++/F1 (50/50) 10 mg/g	–	129.7	29.1	–	–	7
F1++++ (10 mg/g)	BHMW (2.5 mg/g)	183.2	146.9	2.18	2.32	57
F1++++ (10 mg/g)	Bentonite (2.5 mg/g)	199.4	145.6	2.16	2.60	58
F1++++ (10 mg/g)	Bentonite (25 mg/g)	199.4	79.8	2.16	2.05	25
